# Extracellular matrix protein 1 is correlated to carcinogenesis and lymphatic metastasis of human gastric cancer

**DOI:** 10.1186/1477-7819-12-132

**Published:** 2014-04-29

**Authors:** Qiuwan Wu, Xiaohong Li, Haiyan Yang, Chuanhui Lu, Jun You, Zhiming Zhang

**Affiliations:** 1 Xiamen Cancer Centre, the First Affiliated Hospital of Xiamen University (the Teaching Hospital of Fujian Medical University), Xiamen, China

**Keywords:** Extracellular matrix protein 1 (ECM1), Lymphatic microvessel density (LMVD), Gastric cancer, Real-time PCR (RT-PCR), Immunohistochemistry

## Abstract

**Background:**

Tumor-induced lymphangiogenesis is a crucial step in malignant invasion and metastasis. Extracellular matrix protein 1 (ECM1) was recently reported to play a role in lymphangiogenesis. In the present work, we aimed to evaluate the role of ECM1 in gastric cancer and examined whether aberrant expression of ECM1 increased the tumorigenic and metastatic potential of human gastric cancer.

**Methods:**

The mRNA and protein expression of ECM1 in gastric cancer specimen and the noncancerous counterparts from 77 patients were detected by real-time PCR and immunohistochemistry staining. Lymphatic microvessel density (LMVD) in the corresponding serial sections was assessed by counting the lymphatic microvessels labelled by D2-40. The correlations between ECM1 expression, LMVD, and the clinicopathological parameters were examined.

**Results:**

ECM1 protein expression was detected in 70.1% (54/77) of gastric cancer specimen, significantly higher than that in the corresponding counterparts (*P* <0.01). ECM1 mRNA in tumor specimen was also dramatically amplified. Elevated LMVD and ECM1 were positively correlated (*P* <0.01). In addition, ECM1 protein expression was also closely associated with depth of tumor invasion and TNM stage (*P* <0.05, respectively).

**Conclusions:**

ECM1 expression is aberrant elevated in tumor specimen and is closely related to the tumorigenic and metastatic potential of human gastric cancer. Thus, carrying out the protein examination may be beneficial to predict carcinogenesis and metastatic spread of human gastric cancer.

## Background

Tumor metastasis is the main leading cause of cancer-related death in patients with gastric cancer. Lymph node metastasis is the hallmark of tumor progression and is considered as one of the most important prognostic factors. Currently, no effective treatment modalities are available for this progression. Tumor-induced lymphangiogenesis is a crucial step for tumor progression and has been demonstrated to be prior to the onset of lymphatic invasion and metastasis [1-3]. Furthermore, increasing number of lymphatic microvessels provides more opportunities for tumor cells to disseminate to the lymphatic system [4,5]. Nowadays, lymphangiogenesis is quantified by lymphatic microvessel density (LMVD) for convenience, which is applied to evaluate the status of lymphangiogenesis and the remodeling or regression of lymphatic vessels [6-8].

Extracellular matrix protein 1 (ECM1) is a soluble protein, which was first identified in 1994 [9]. Mutations in this gene were reported to be associated with lipoid proteinosis disorder (known as hyalinosis cutis et mucosae or Urbach-Wiethe disease), which is characterized by generalized thickening of skin, mucosa, and certain viscera [10]. Recent study revealed that a homozygous frameshift mutation in ECM1 led to a failure of human mucocutaneous lymphangiogenesis [11], suggesting the role of this gene in the progression of lymphangiogenesis. It was reported that highly purified recombinant ECM1 stimulated proliferation of cultured endothelial cells [12]. Our previous study found that both ECM1 and vascular endothelial growth factor-C (VEGF-C) have a synergistic effect on lymphangiogenesis, so as to facilitate lymphatic metastasis of breast cancer [13]. However, whether ECM1 correlates to carcinogenesis and metastasis of gastric cancer has not yet been clarified.

Therefore, we have attempted to determine the relationship between ECM1 expression, LMVD, and the clinicopathological parameters in gastric cancer patients to further discuss the clinical significance of ECM1 in carcinogenesis and lymphatic metastasis of human gastric cancer.

## Methods

### Patients and specimens

The research protocol was approved by the Ethics Committee of Human Experimentation in our country and was performed in accordance with the principles of the Declaration of Helsinki. All specimens were obtained from patients who had undergone gastrectomy at our cancer center between 2009 and 2010. Primary tumor specimen and the matched non-cancerous mucosal tissue (located more than 5 cm away from tumor margins) were collected. All diagnosis has been confirmed by pathology. Metastatic tumor specimen from other tissue origins or from patients underwent neoadjuvant chemotherapy or radiotherapy was excluded. Written informed consents were obtained from all patients.

### Real-time PCR (RT-PCR)

Specimens were frozen immediately in liquid nitrogen after being isolated. Total RNA was extracted from the frozen tissues using Trizol reagent according to the manufacturer’s instruction (Invitrogen, USA). Reverse transcription of total RNA into cDNA was conducted using TaKaRa Reverse Transcription Reagents (TaKaRa Bio, Japan) at 37°C for 15 min, followed by 85°C for 5 s. Primers were designed using Primer Premier 5.0 software (Premier, Canada) and synthesized by Invitrogen, USA. Each reaction was performed in triplicate. The oligonucleotide primers were as follows: *ECM1* mRNA sequence-specific primers (GenBank: NM 004425.3) were forward: 5′-CAAATCTGCCTTCCTAACCG-3′ and reverse: 5′-AAGCAGGAGAACCGAGCC-3′; *GAPDH* mRNA was used as an internal standard. Its mRNA sequence-specific primers (GenBank: NM 002046.3) were forward: 5′-GAAGGTGAAGGTCGGAGTC-3′ and reverse: 5′-GAAGATGGTGATGGGATTTC-3′. Real-time quantitative PCR was performed using the TaKaRa SYBRR® Premix Ex Taq™ II PCR kit (TaKaRa Bio, Japan) in a Roche Lightcycler 480 instrument (Roche, Switzerland). Reactions were performed in 10 μL volumes with denaturation at 95°C for 5 s, annealing at 58°C for 15 s, and extension at 72°C for 20 s, over 45 cycles. Semi-quantitative analysis was conducted by averaging the triplicates of the cycle threshold (Ct) for the target genes and dividing the average of the triplicate obtained from GAPDH in the same specimen.

### Immunohistochemistry staining and evaluation

Briefly, 4 μm consecutive sections of formalin-fixed, paraffin-embedded tissues were deparaffinized, stepwise rehydrated, and the endogenous peroxide blocked. Before staining, slides were performed using microwave antigen retrieval, and then incubated with anti-ECM1 antibody (clone SC-05, Abcam, UK; 1:20 dilution) and D2-40 antibody (clone D2-40, Abcam, UK; 1:40 dilution), respectively. Non-specific binding was blocked by using 10% non-immune serum (Santa Cruz, CA, USA.) for 10 min prior to antibody incubation. After rinsing, slides were incubated with EnVision™ Detection Systems (Dako, Denmark), counterstained with hematoxylin, dehydrated, and mounted. Negative controls were processed using the same procedure, except that 10% non-immune mouse serum (Santa Cruz, CA, USA.) was used in place of the primary antibodies. All sections were also stained by hematoxylin and eosin (H&E) to confirm their histological diagnosis and other microscopic characteristics. Tumor size, depth of invasion, and lymph node metastasis were determined by pathology.

Morphometric analyses and staining scores were estimated independently by two observers who had no prior knowledge of the patients’ clinicopathologic data. As previously reported [7], a modified Weidner’s method was employed to calculate lymphatic microvessel density (LMVD). It was assessed by counting the number of immunostained vessels with D2-40 staining on tissue sections. The area containing the most stained vessels (‘hot spots’) was first identified by scanning the whole section at low magnification (×40); then number of positive vessels was counted in two high magnification fields (×200) in the hot spot. LMVD in tumor sections was determined by averaging the number of total lymphatic vessels in all the fields of each slide, including within the tumor or at the periphery of the tumor. The number of visible microvessels (LMVD) was calculated as the average of four counts (two authors and two microscopic fields). If there was any discrepancy of more than 10% of the microvessel count, these discordant sections were recounted until reaching consensus. ECM1 expression was judged according to the methods previously described [14]: the percentage of positive staining = (the numbers of positive samples/the numbers of samples tested) × 100%.

### Statistical analysis

SPSS (Chicago, IL, USA) software was employed. Data which were normally distributed were expressed as mean ± standard error of the mean (S.E.M.). Statistical evaluation was performed using Spearman correlation test to analyze the rank data and Mann–Whitney *U*-test to differentiate non-parametric means of different groups. Chi-square test, Yates’ correction, or Fisher’s exact test was used to analyze qualitative independent variables. All statistical tests were two-side. A *P* value of less than 0.05 was considered as statistically significant.

## Results

### LMVD counting

**Figure 1:**
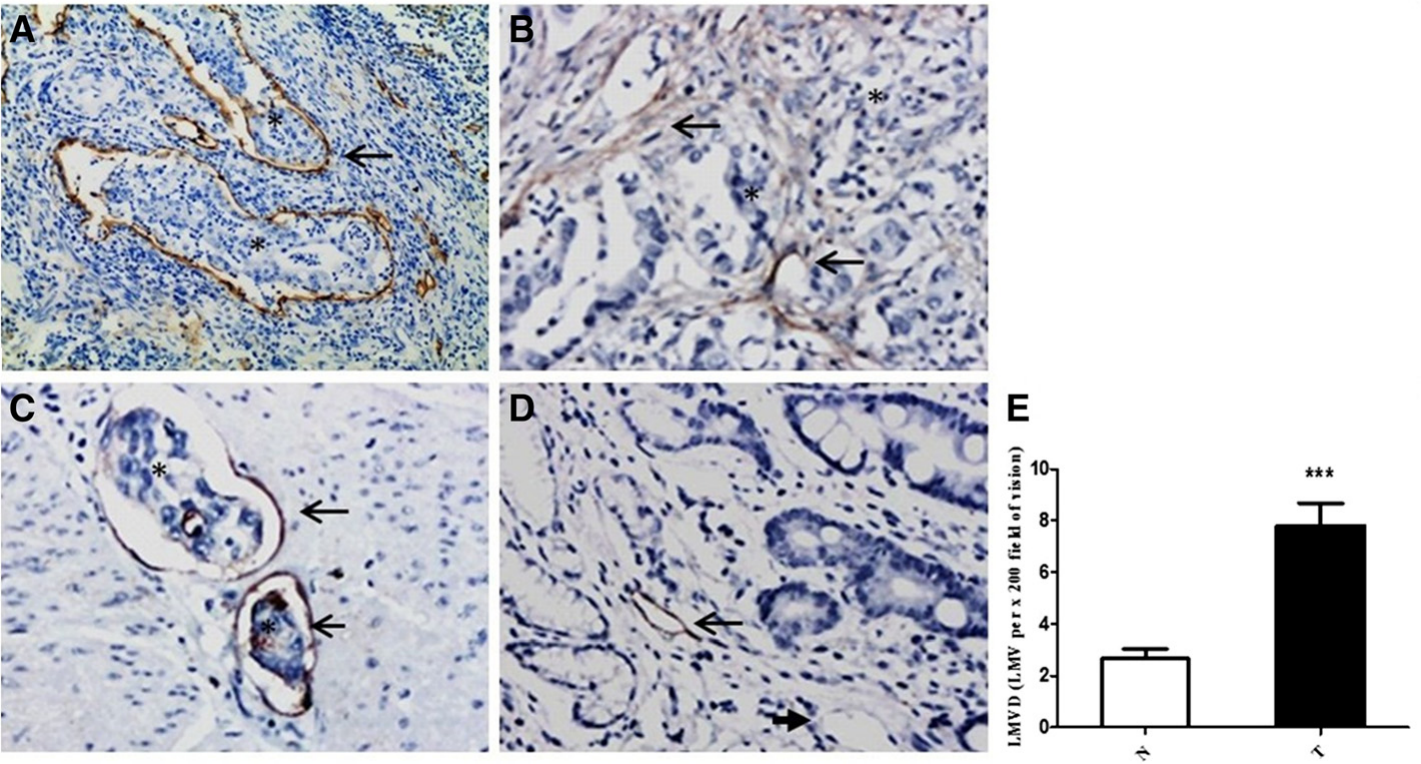
**Immunohistochemical labeling of lymphatic microvessels (EnVision™) and LMVD in primary gastric cancer tissue and noncancerous gastric tissue. (A-D)** Lymphatic microvessels labeled with D2-40 by IHC (EnVision™). **(A, B)** Gastric cancer (×100), (A) peritumoral lymphatic vessels were large, within which was nests of no staining tumor cells (asterisks); (B) intratumoral lymphatic vessels had narrow and collapsed lumen, with irregular cell walls (long arrow). **(C)** Invaded muscle tissue (×200). There were a few tumor cells in dilated lymphatic microvessels, as ‘lymphatic vessel invasion’ (asterisks). **(D)** Non-cancerous mucosa (×200). Long arrows indicated lymphatic microvessels, and short arrows denoted blood capillaries. Asterisks represented the areas where tumor cells are. **(E)** LMVD in gastric cancer tissue (T) was statistically higher than that in the non-cancerous counterparts (N) (*** *P* <0.001).

D2-40 is a highly specific marker for lymphatic endothelium and has been proven valuable in distinguishing lymph vessels from blood vessels [15-17]. Microscopically, immunostained lymphatic vessels were lined with a single layer of endothelial cells, but the adjacent blood vessels showed negative staining (Figure 1A-D). LMVD in gastric cancer tissue from 77 patients was 7.79 ± 0.88 lymphatic microvessels per × 200 field (LMV per × 200 field). While in the non-cancerous counterparts, LMVD was 2.65 ± 0.38 LMV per × 200 field. The difference in LMVD between these two tissue types was statistically significant (Mann–Whitney test, *P* <0.001) (Figure 1E).

**Figure 2:**
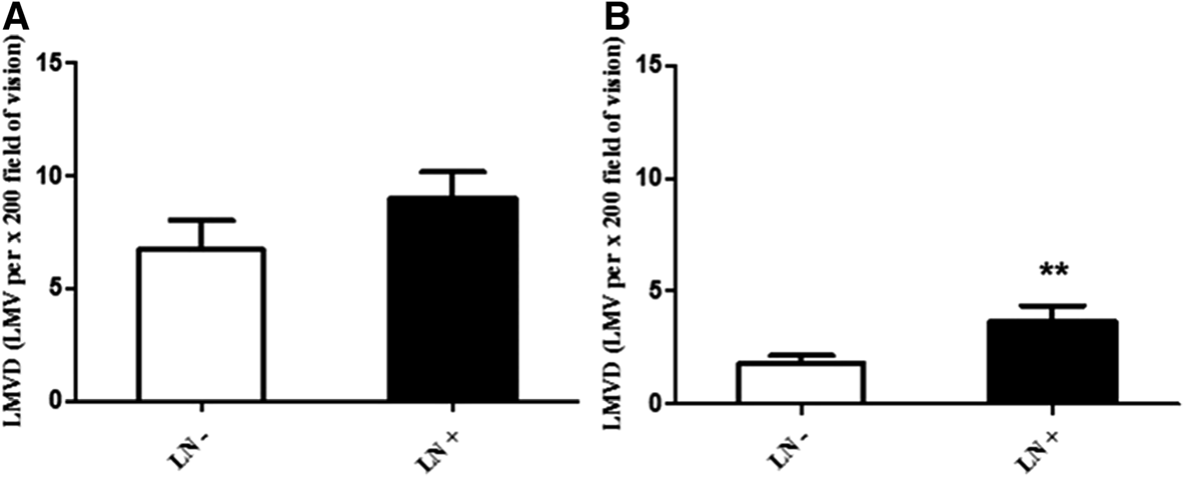
**Comparison of LMVD in tissues without/with lymph node metastasis. (A)** LMVD in tumor specimen with lymph node metastasis (LN+) was higher than that without lymph node metastasis (LN-), although the difference did not reach statistical significance (*P* >0.05). **(B)** LMVD in non-cancerous gastric mucosa from patients with lymph node metastasis (LN+) was statistically higher than that without metastasis (LN-) (** *P* <0.01).

As shown in Table 1, there was no statistical difference of LMVD among groups of different gender, age at diagnosis, differentiation degree, and depth of tumor invasion, lymph node metastasis or TNM stage (*P* >0.05, respectively). In addition, though the difference was not statistically significant (Mann–Whitney test, *P* = 0.065), LMVD in the tumor specimen with lymphatic metastasis (9.02 ± 1.16 LMV per × 200 field) tended to be higher than that without lymph node metastasis (6.74 ± 1.29 LMV per × 200 field) (Figure 2A). And LMVD in paracancerous gastric mucosa with lymph node metastasis (3.66 ± 0.70 LMV per × 200 field) was statistically higher than that without metastasis (1.79 ± 0.34 LMV per × 200 field; Mann–Whitney test, *P* = 0.008) (Figure 2B).

**Table 1:** ECM1 expression, LMVD, and clinicopathological characteristics

**Clinicopathological parameters**	**N**		**ECM1 expression**		**LMVD**	
		**-**	**+**	***P***	**(LMV per × 200 field)**	***P***
*Gender*				0.618		0.627
Male	47	13	34		7.76 ± 1.21	
Female	30	10	20		7.83 ± 1.23	
*Age (years)*				0.628		0.553
<60	37	10	27		7.25 ± 1.17	
≥60	40	13	27		8.34 ± 1.33	
*Differentiation degree*				0.489		0.253
Low	36	11	25		9.25 ± 1.37	
Moderate	23	5	18		5.75 ± 1.22	
Well	18	7	11		7.94 ± 2.01	
*Depth of invasion*				<0.0001***		0.721
T1-T2	23	18	5		8.77 ± 1.97	
T3-T4	54	5	49		7.53 ± 0.99	
*Lymph node metastasis*				0.521		0.110
0	34	9	25		6.74 ± 1.29	
1-2	18	9	11		7.00 ± 2.46	
3-6	10	3	7		11.00 ± 2.74	
≥7	15	4	11		9.57 ± 1.47	
*TNM stage*				0.0014**		0.241
0-I	21	12	9		8.50 ± 2.12	
II-III	27	11	45		8.67 ± 1.18	

‘+’, ‘++’, and ‘+++’ for ECM1 IHC staining are grouped together as ‘+’.

T1: lamina propria and submucosa; T2: muscularis propria and subserosa; T3: exposure to serosa; T4: invasion into serosa.

**P <0.01.

***P <0.001.

*P* <0.05 is considered statistically significant.

### ECM1 expression

**Figure 3:**
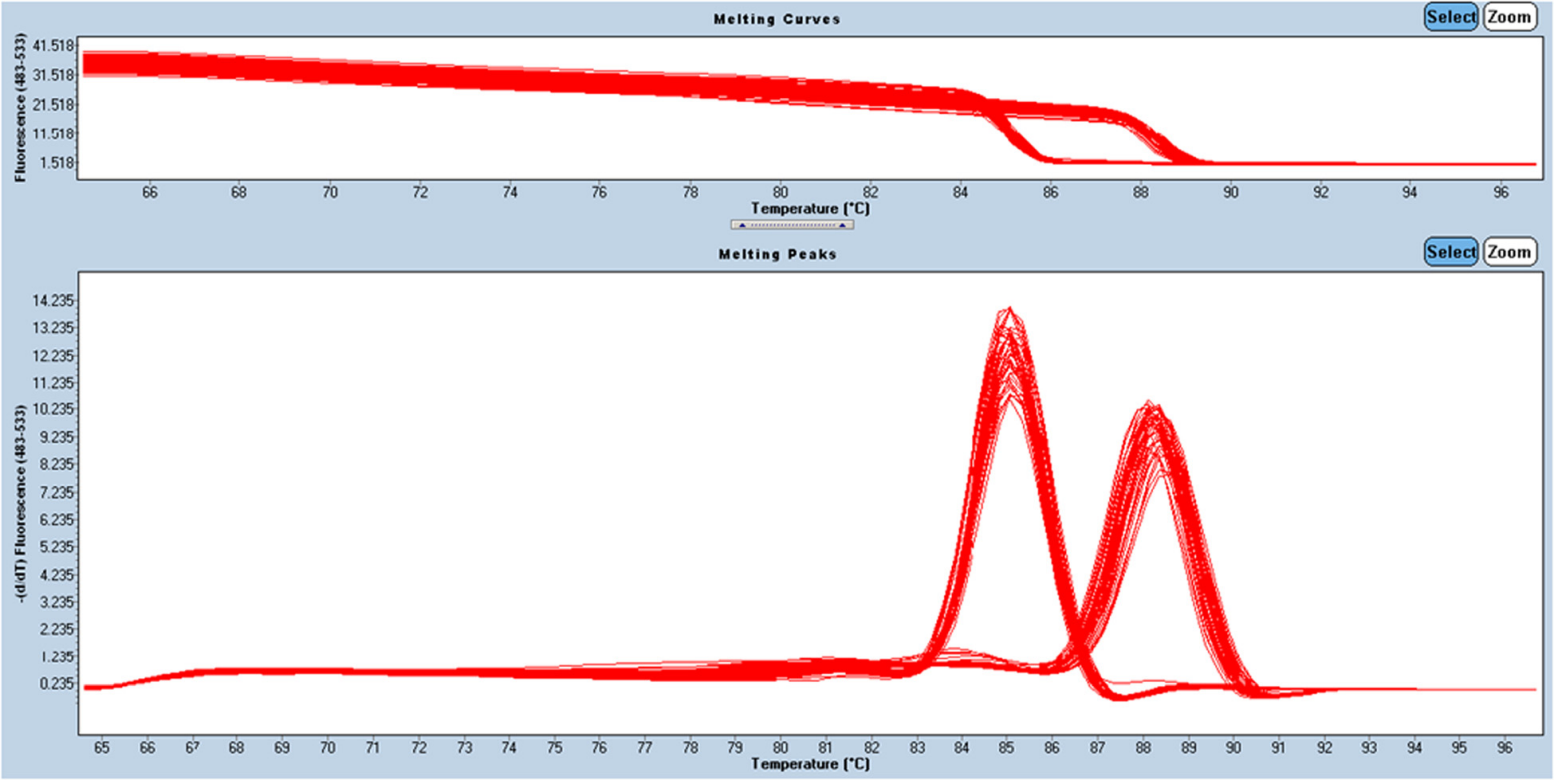
**Melting curves of GAPDH and ECM1 real-time fluorescent quantitative PCR products (T**_**m **_**of GAPDH is 85.05°C, while that of ECM1 is 88.3°C).**

**Figure 4:**
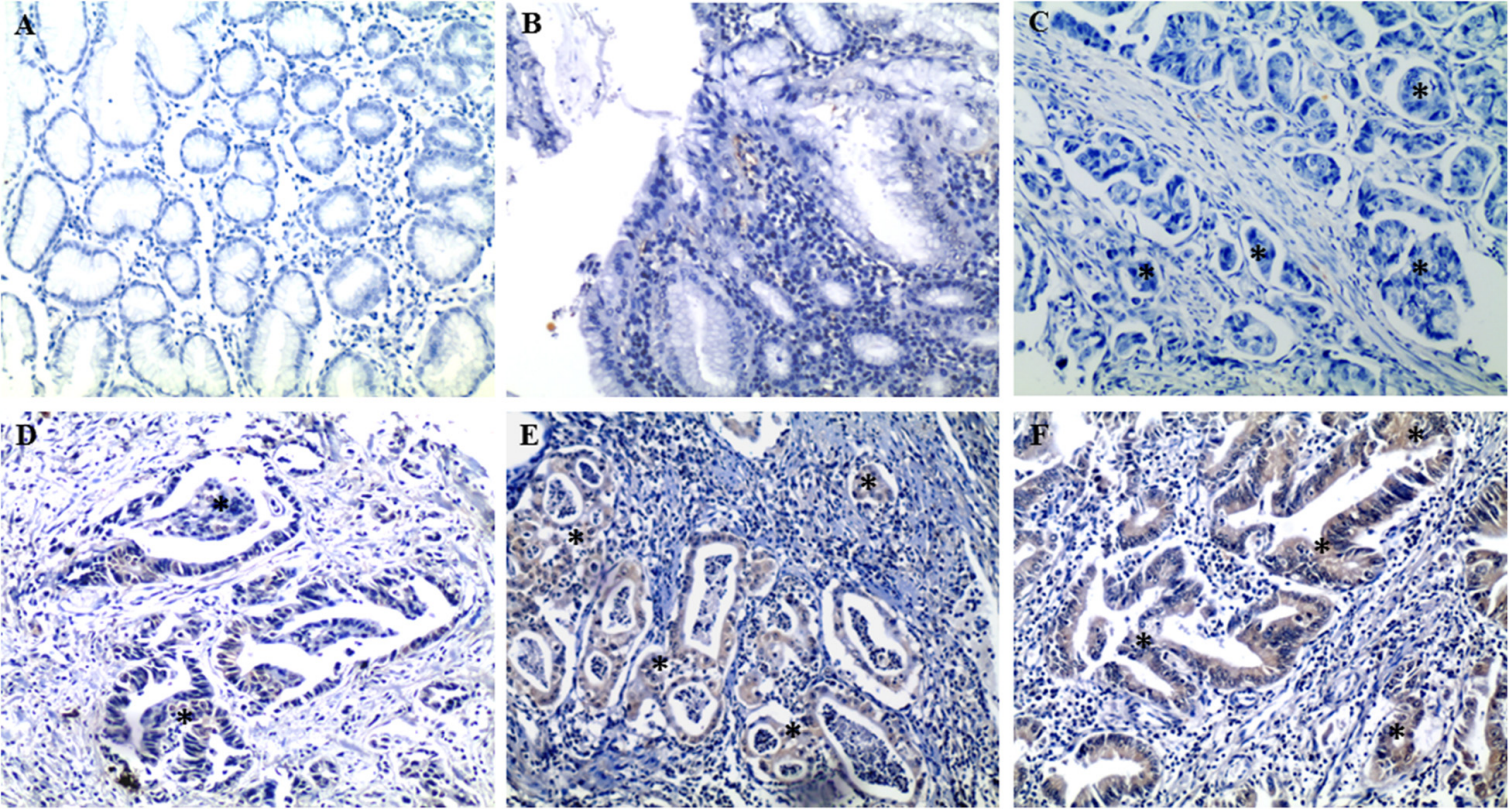
**Representative IHC staining of ECM1 (EnVision™, ×100). (A)** and **(C)** negative for ECM1 staining, **(B)** and **(D)** + for ECM1 staining, **(E)** ++ for ECM1 staining, **(F)** +++ for ECM1 staining. Asterisks represent the areas where tumor cells are. **(A, B)** non-cancerous gastric mucosa, **(C, D)** gastric cancer, **(E, F)** metastatic lymph node.

The corresponding melting curves of PCR products were shown in Figure 3. The mean normalized expression level of ECM1 mRNA in tumor specimen was statistically higher than that in non-cancerous counterparts (Mann–Whitney test, *P* <0.01; Table 2). ECM1 protein was specifically expressed in the cytoplasm of tumor cells, with scattered expression in cell membrane (Figure 4); whereas, the nucleus had no staining. Notably, the protein was also detected in the cytoplasm of metastatic cells in invaded tissue and lymph node (Figure 4E, F). The difference in the rate of ECM1 expression between tumor tissue (54/77, 70.1%) and the non-cancerous counterparts (6/77, 7.8%) was statistically different (*χ*^2^ = 62.91, *P* <0.001; Table 2). As shown in Table 1, the protein expression of ECM1 in tumor specimen was correlated with depth of tumor invasion and TNM stage (Fisher’s exact test, *P* <0.001 and *P* = 0.002, respectively). However, correlation of ECM1 expression with other clinicopathologic characteristics was not significant, including patients’ gender, age at diagnosis, differentiation degree, or lymph nodes metastasis (*P* >0.05, respectively). And ECM1 expression rate in tumor specimen with lymph node metastasis (25/34, 73.5%) was not statistically higher than that without lymph node metastasis (29/43, 67.4%) (Fisher’s exact test, *P* = 0.623).

**Table 2:** ECM1 expression in gastric cancer specimen and non-cancerous counterparts

**Tissue type**	**mRNA level of ECM1**	**F**	**Positive rate (%)**	**Staining grades of ECM1**				***χ***^**2**^
				**-**	**+**	**++**	**+++**	
Tumor specimen	0.010 ± 0.002		70.1(54/77)	23	35	13	6	
Non-cancerous mucosa	0.007 ± 0.002	7.48**	7.8(6/77)	71	5	1	0	62.91***

**P <0.01.

***P <0.001.

*P* <0.05 is considered statistically significant.

### ECM1 expression and LMVD

**Figure 5:**
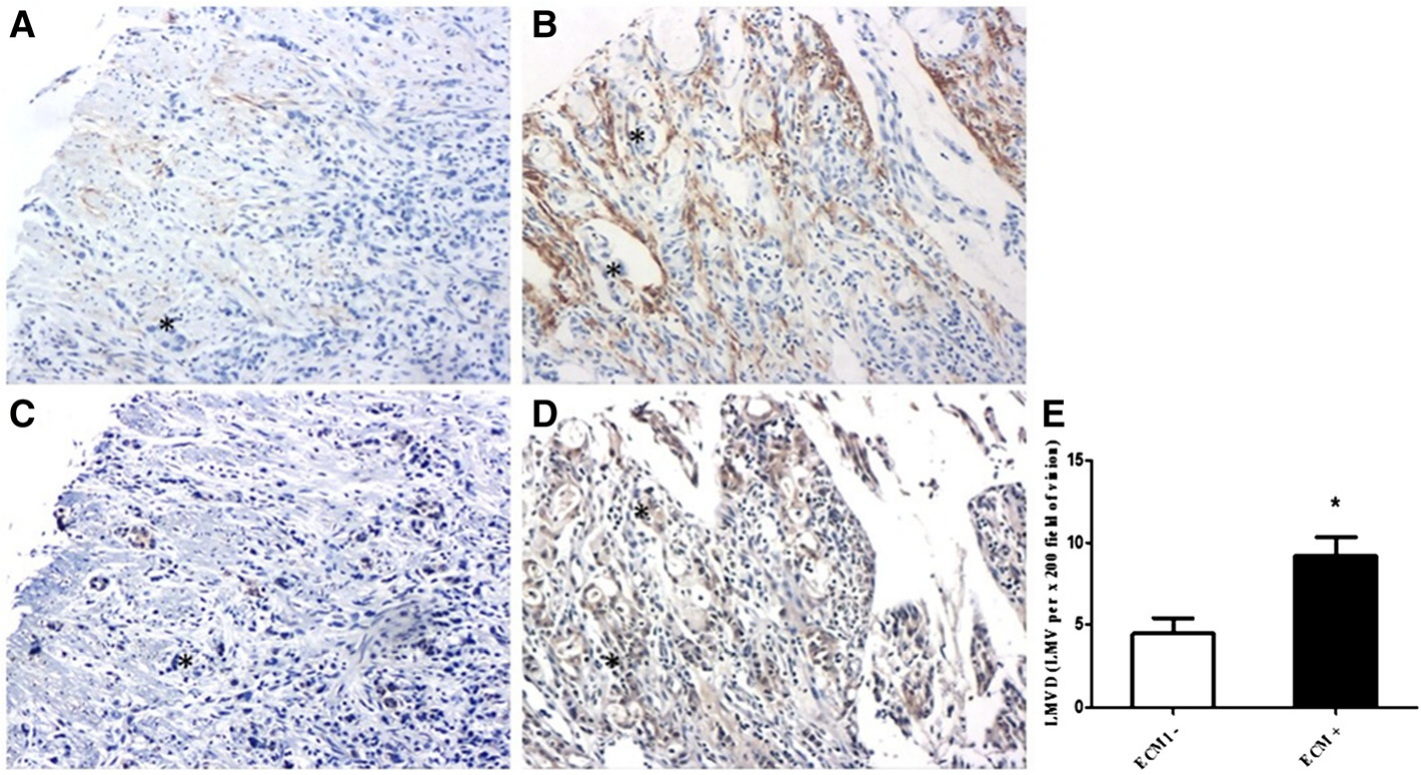
**Correlation of LMVD and ECM1 expression in gastric cancer (EnVision™, ×100). (A, B)** lymphatic microvessels labelled by D2-40, **(C)** +/- for ECM1 staining, **(D)** +++ for ECM1 staining. **(A, C)** &**(B, D)** represent the matched specimen from the same patient, respectively. Asterisks represent the areas where tumor cells are. **(E)** LMVD in gastric cancer with ECM1 positive staining (ECM1+) was statistically higher than that without ECM1 staining (ECM1-) (* *P* <0.05).

As representative shown in Figure 5, tumor sections which were ECM1-positive staining had elevated LMVD (Mann–Whitney test, *P* = 0.014). A positive correlation was further established between ECM1 expression and LMVD in gastric cancer tissue (Spearman’s R = 0.407, *P* = 0.001; Table 3).

**Table 3:** ECM1 expression and LMVD (LMV per x 200 field) in gastric cancer and non-cancerous counterparts

**Tissue type**	**ECM1 immunostaining**		**Spearman R**	***P***
	**-**	**+**		
Tumor specimen	(23/77) 4.47 ± 0.95	(54/77) 9.22 ± 1.13	0.407	0.001**
Non-cancerous mucosa	(71/77) 2.71 ± 0.41	(6/77) 2.00 ± 1.05	-0.061	0.633

‘+’, ‘++’, and ‘+++’ for ECM1 IHC staining are all grouped together as ‘+’.

**P <0.01 is considered statistically significant.

## Discussion

Lymphangiogenesis plays an essential part in carcinogenesis and metastasis of malignancies, as newly born lymph microvessels are the direct access to lymph nodes and distant spread. Our present study showed that, compared to the non-cancerous counterparts, the number of lymphatic microvessels obviously increased in gastric cancer. Lymphatic microvessels are well-known to be a discontinuous basement membrane and lack tight interendothelial junctions. Therefore, it is believed that these vessels would be convenient for tumor cells to pass through. It is meant that, tumor-induced lymphangiogenesis provides more opportunity for tumor cells to the lymphatic system and even distant dissemination [4,5,18].

As a tumor-derived protein, ECM1 was previously reported to be over-expressed in various malignant epithelial tumors, and tumors with lymph node metastases were more likely to be ECM1-positive [13,19,20]; although the exact role of this protein was still controversial. It was known that tumor-induced lymphangiogenesis was prior to the onset of lymphatic metastasis in lymph node and distant spread [1-3]. In the present study, that ECM1 over-expression was positively correlated with increasing number of lymph microvessels in tumor specimen suggest the potential value of this protein in predicting lymphatic metastasis of gastric cancer. Since ECM1 plays a vital role in the promotion for endothelial cells proliferation [12] and lymphangiogenesis [13], we presumed that the protein might involve in lymphangiogenesis participating in the metastatic progression of gastric cancer. Moreover, the same as in the primary tumor, ECM1 was also expressed in metastatic cells of invaded tissues, indicating that the protein was correlated with metastatic potential of tumor cells. Would this provide a clue of the prediction of ECM1 in carcinogenesis and metastasis of gastric cancer? Of course, further studies are still needed to further determine.

Compared to the non-cancerous counterparts, both mRNA and protein expression of ECM1 were elevated in gastric cancer tissue. Moreover, the protein was positively correlated with depth of tumor invasion and TNM stage. Though a previous study showed that the level of ECM1 mRNA expression was higher in TNM stage I thyroid cancers than in stage II and III tumors [20]. Our results are consistent with the ECM1 expression status observed in hepatocellular cancer [19] and suggest that ECM1 expression correlates to carcinogenesis and invasiveness of tumor cells. Different specimens from variant tissues and relative small sample size may cause inconsistencies in the results obtained from these studies. It was also reported that ECM1 could be an important prognostic marker in patients with breast cancer [21] or with hepatocellular cancer [19], which indicated that detecting abnormal expression of this protein would be useful to predict an unfavorable prognosis in malignancy.

## Conclusions

In summary, ECM1 expression is aberrant elevated in gastric cancer and is positively correlated with LMVD and several clinicopathological characteristics, as is depth of tumor invasion and TNM stage; thus providing a clue that evaluation of ECM1 expression in gastric cancer tissues facilitates the prediction of carcinogenesis and metastatic spread in human gastric cancer. We hope that this report would facilitate to manage the disease and benefit the patients in clinical practice.

## Competing interests

The authors declare that they have no competing interests.

## Authors’ contributions

QW and XL drafted the manuscript and participated in all other parts of the work. QW performed the immunoassays. XL and HY performed the molecular studies. HY and CL performed the statistical analyses. JY and ZZ conceived the study, participated in its design and coordination, and helped to draft the manuscript. All authors read and approved the final manuscript.
